# A Case of a Pregnant Woman with Thrombosis in an Artificial Aortic Valve Resulting in Severe Cerebral Hemorrhage in the Newborn

**DOI:** 10.1155/2018/6154382

**Published:** 2018-07-29

**Authors:** Hidetake Kamei, Yu Wakimoto, Yumi Murakami, Maya Omote, Kayoko Harada, Atsushi Fukui, Hiroyuki Tanaka, Hideaki Sawai, Hiroaki Shibahara

**Affiliations:** Department of Obstetrics and Gynecology, Hyogo College of Medicine, 1-1 Mukogawa-cho, Nishinomiya, Hyogo 663-8501, Japan

## Abstract

Many patients, after artificial valve replacement surgery, receive warfarin anticoagulant therapy. However, it has been reported that warfarin administration during pregnancy can cause fetal teratogenicity. With reference to this case, we will discuss how warfarin administration in mid-pregnancy caused severe cerebral hemorrhage in the newborn child. The 36-year-old patient in this case underwent aortic valve replacement surgery when she was 11 years old; this requires the continued use of warfarin after surgery. Although she was advised otherwise, the patient became pregnant. The warfarin treatment was discontinued at 5 weeks of gestation and she began self-injection of heparin; however, her health quickly deteriorated requiring an emergency, warfarin treatment. On gestation week 21, she was admitted to our hospital with a high likelihood of a spontaneous abortion. A week later, transesophageal ultrasonography revealed a thrombus in the patient's aortic valve. Because of this finding, we re-started warfarin administration. At 32 weeks of gestation, cardiotocography showed decreased fetal heart rate; thus, an emergency Cesarean section was performed. A baby was delivered, weighing 1,702 g with an Apgar Score of 1 at 1 minute and 4 at 5 minutes. Cranial computed tomography of the infant showed bilateral intraventricular hemorrhage and ventricular dilation. In order to protect the mother and prevent hemorrhage in the newborn, it is recommended that a continuous heparin infusion should be administered to the pregnant woman after the 36th week of gestation. Regarding the impact on the infant, it is considered that continuous intravenous administration of heparin is safer during the third trimester of pregnancy. However, administration of heparin alone makes the preventive effect of thrombosis uncertain. When warfarin is administered in pregnancy, pregnancy management should be performed bearing the risk of fetal cerebral hemorrhage in mind.

## 1. Introduction

Recent advances in cardiac surgical techniques have enabled women to carry pregnancy to full term [1]. However, there are risks involved; thus, continuous perinatal care is required. We report a case of the perinatal management of a patient with aortic valve thrombosis occurring after mechanical valve replacement.

## 2. Case

**Figure 1:**
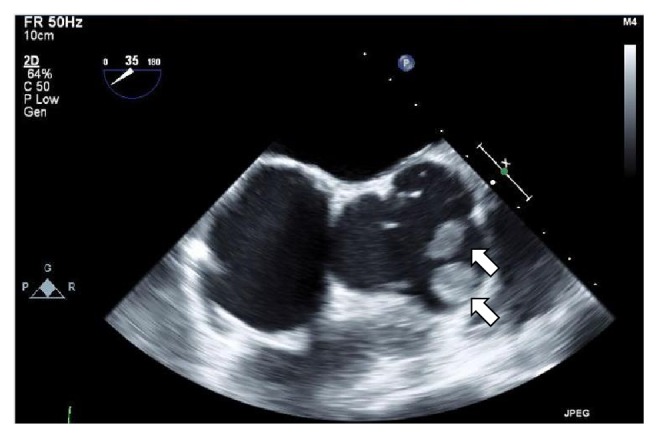
Transesophageal echocardiography. A transesophageal echocardiography of the mother's heart showed a movable thrombus on the aortic valve at 22 weeks of gestation. The size of the biggest thrombus was 26 × 8 mm (arrows).

**Figure 2:**
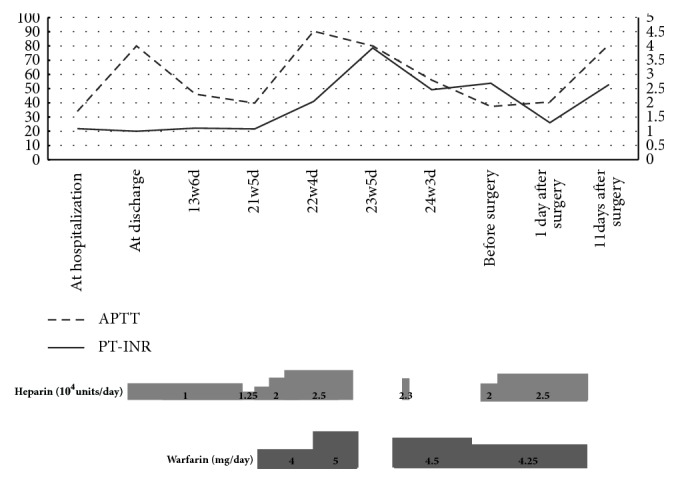
The change of APTT and PT-INR. This figure shows the change of APTT and PT-INR in the mother's blood in relation to dosage of heparin and warfarin.

The patient was a 36-year-old gravida 0 woman. At the age of 7, she underwent ventricular septal defect closure for the right ventricular outflow tract. At the age of 11, she received a mechanical aortic valve replacement. Since after the replacement, she has been receiving warfarin orally at a dosage of 4.5 mg/day. She conceived naturally and she was referred to our hospital for perinatal management. Oral administration of warfarin was discontinued at 5 weeks of gestation and she began self-injection of heparin. At 21 weeks and 5 days of gestation, she was admitted to our hospital with a high risk of spontaneous abortion and was put on intravenous ritodrine. This successfully prevented a miscarriage. At 21 weeks and 6 days of gestation, we started a continuous infusion of 25,000 units of heparin daily. On the 22nd week, transesophageal echocardiography showed a movable thrombus in the aortic valve. The size of the biggest thrombus was 26 × 8 mm (Figure 1). We increased the dosage of heparin to 28,000 units daily and restarted the administration of warfarin. Following this, the thrombus reduced in size, and at 23 weeks and 5 days transesophageal echocardiography showed no signs of thrombosis in the patient. At 32 weeks and 2 days of gestation, a routine cardiotocography showed a decreased fetal heart rate; thus, an emergency Cesarean section was performed under general anesthesia because of the presence of warfarin in the blood. The baby was delivered, weighing 1,702 g, with an Apgar Score of l at l minute, and 4 at 5 minutes. The total amount of blood loss during the surgery was 1,410 ml. During the surgery, 16 units of fresh frozen plasma (FFP) was transfused; and after surgery, we continued to infuse 20,000 units of heparin daily. On the 11th day after surgery, owing to continuous genital bleeding, heparin administration was discontinued and uterine artery embolization was performed. This treatment stopped the bleeding and on the 21st postsurgical day; we started warfarin administration at 5 mg/day. She was discharged on the 34th postoperative day due to the stable PT-INR levels (Figure 2).

**Figure 3:**
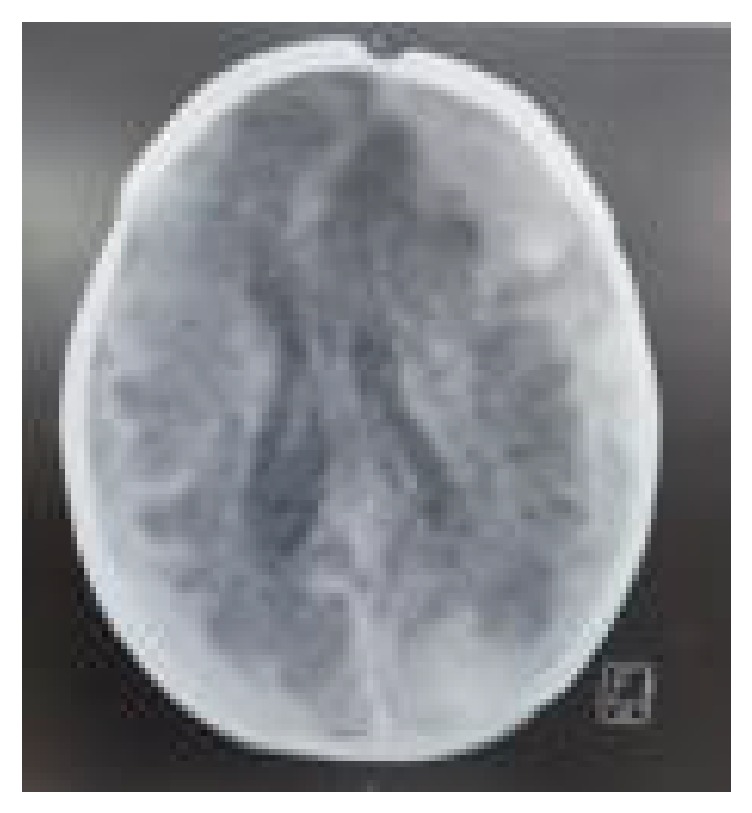
Cranial CT of the newborn. Cranial CT scan of the newborn showed bilateral intraventricular hemorrhage with ventricular dilatation and midline shift.

The newly born infant was intubated and admitted to the newborn intensive care unit. At the time of admission, activated partial thromboplastin time was 180 seconds or more and bilateral intracerebral ventricular hemorrhage was detected using ultrasonography. On the first day of life, anemia was observed in the infant and red cell concentrate and FFP were transfused (Table 1). We attempted to reduce the infant's dependence on the ventilator and at 8 days of age the infant was extubated. On the postnatal 10th day, a cranial CT scan showed bilateral intraventricular hemorrhage with ventricular dilation and midline shift (Figure 3). Although convulsions accompanying the intracranial hemorrhage were observed, the infant's general condition was stable and oral feeding was started on postnatal day 10. The newborn was discharged on postnatal day 54. However, the infant later developed cerebral palsy and is currently receiving treatment at our hospital.

## 3. Discussion

In pregnant women who have undergone artificial valve replacement surgery, mortality rate is high for both mother and fetus during pregnancy. Treatment requires perinatal care with special attention to the possible onset of thromboembolism [2, 3]. During late pregnancy, fibrinogen, von Willebrand factor, factors VIII, IX, X, and XII, are increased and activated; thus, the risk of thrombosis and embolism increases [4]. Therefore, continuous assessment of this risk is needed for women in the later stage of pregnancy with mechanical valves [5]. For a woman who wishes to have a baby after aortic valve replacement surgery, a biological valve is often selected, but in some women, a mechanical valve is used, to reduce the possibility of reoperation due to ageing and, thus, the deterioration of a biological valve [6, 7]. In this case, the aortic valve replacement surgery was performed at the age of 11 years and mechanical valves were used. For pregnant women with mechanical valves, it is reported that the dosage of the anticoagulant should be adjusted so that APTT is 1.5 to 2.5 times the normal value. Moreover, it is reported that the effect of heparin will result in a vast change in APTT; therefore, strict monitoring is necessary. In this case, autologous injection of heparin began from 5 weeks of gestation, and continuous infusion of heparin started from 21 weeks of gestation. APTT was measured twice a week and the values of around 40-80 seconds were maintained, but several thrombi appeared on the mechanical valve at 22 weeks of gestation. It has been reported that the incidence of thrombosis in pregnant women with mechanical valves was 3.9% in the warfarin continuation group, 9.2% in the warfarin use after unfractionated heparin use for 12 weeks of pregnancy group, and 25% in the unfractionated heparin use throughout pregnancy group [8]. Comparing between heparin and warfarin anticoagulant therapy during pregnancy, thrombosis occurred significantly in the heparin group, ranging from 12 to 24 percent, indicating that serious complications may occur [9]. Therefore, in this case, because of the high risk of developing maternal cerebral infarction, warfarin administration was started after informing the patient about the side effects of abnormal blood coagulation in the fetus. Thus, after restarting warfarin administration, the multiple thrombi on the mechanical valve disappeared, but intraventricular hemorrhage appeared in the newborn. Blood collection data at the birth showed an abnormally high PT-INR value; the enzyme system of the fetus was underdeveloped and vitamin K-dependent coagulation factor was lower than the normal range. The influence of warfarin was apparent. In Japan, pregnant women with artificial valves are permitted to use warfarin for thrombus treatment during pregnancy in life-threatening circumstances [10]. However, as anticoagulation therapy and antiplatelet therapy during pregnancy affect not only the mother but also the infant, it is necessary to perform pregnancy management, bearing in mind the risk of fetal cerebral hemorrhage during the perinatal management.

## Figures and Tables

**Table 1:** Blood sample from neonate immediately after birth and at 1 day of age.

**Postpartum**	**Day 0**	**Day 1**
AT-3(%)	70	72

D-dimmer (*μ*g/ml)	1.2	1.5

Fibrinogen (mg/dl)	553	621

APTT (seconds)	≧180	37.8

PT-INR	Unmeasurable	1.02

Platelet (× 10^4^/*μ*l)	50.3	53.2

Hb (g/dl)	6.8	7.1
